# Carotenoid metabolism during bilberry (*Vaccinium myrtillus* L.) fruit development under different light conditions is regulated by biosynthesis and degradation

**DOI:** 10.1186/s12870-016-0785-5

**Published:** 2016-04-21

**Authors:** Katja Karppinen, Laura Zoratti, Marian Sarala, Elisabete Carvalho, Jenni Hirsimäki, Helmi Mentula, Stefan Martens, Hely Häggman, Laura Jaakola

**Affiliations:** Genetics and Physiology Unit, University of Oulu, P.O. Box 3000, FI-90014 Oulu, Finland; Climate laboratory Holt, Department of Arctic and Marine Biology, UiT the Arctic University of Norway, NO-9037 Tromsø, Norway; Fondazione Edmund Mach, Research and Innovation Center, via E. Mach 1, 38010, San Michele all’Adige, TN Italy; NIBIO, Norwegian Institute of Bioeconomy Research, P.O. Box 115, NO-1431 Ås, Norway

**Keywords:** *Vaccinium*, Carotenoid biosynthesis, Berry ripening, Lutein, Beta-carotene, Gene expression, Red light

## Abstract

**Background:**

Carotenoids are important pigments and precursors for central signaling molecules associated in fruit development and ripening. Carotenoid metabolism has been studied especially in the climacteric tomato fruit but the content of carotenoids and the regulation of their metabolism have been shown to be highly variable between fruit species. Non-climacteric berries of the genus *Vaccinium* are among the best natural sources of health-beneficial flavonoids but not studied previously for carotenoid biosynthesis.

**Results:**

In this study, carotenoid biosynthetic genes, *PSY, PDS, ZDS, CRTISO, LCYB, LCYE, BCH* and *CYP450-BCH*, as well as a carotenoid cleavage dioxygenase *CCD1* were identified from bilberry (*V. myrtillus* L.) fruit and their expression was studied along with carotenoid composition during fruit development under different photoperiod and light quality conditions. Bilberry was found to be a good source of carotenoids among fruits and berries. The most abundant carotenoids throughout the berry development were lutein and *β*-carotene, which were accompanied by lower amounts of 9Z-*β*-carotene, violaxanthin, neoxanthin, zeaxanthin, antheraxanthin and *β*-cryptoxanthin. The expression patterns of the biosynthetic genes in ripening fruits indicated a metabolic flux towards *β*-branch of the carotenoid pathway. However, the carotenoid levels decreased in both the *β*-branch and *ε*,*β*-branch towards bilberry fruit ripening along with increased *VmCCD1* expression, similarly to *VmNCED1*, indicating enzymatic carotenoid cleavage and degradation. Intense white light conditions increased the expression of the carotenoid biosynthetic genes but also the expression of the cleavage genes *VmCCD1* and *VmNCED1,* especially in unripe fruits. Instead, mature bilberry fruits responded specifically to red/far-red light wavelengths by inducing the expression of both the carotenoid biosynthetic and the cleavage genes indicating tissue and developmental stage specific regulation of apocarotenoid formation by light quality.

**Conclusions:**

This is the first report of carotenoid biosynthesis in *Vaccinium* berries. Our results indicate that both transcriptional regulation of the key biosynthetic genes and the enzymatic degradation of the produced carotenoids to apocarotenoids have significant roles in the determination of the carotenoid content and have overall effect on the metabolism during the bilberry fruit ripening.

**Electronic supplementary material:**

The online version of this article (doi:10.1186/s12870-016-0785-5) contains supplementary material, which is available to authorized users.

## Background

Fruits and berries are important components of the human diet providing a source of many nutritive and bioactive compounds such as carotenoids [1, 2]. The bright red and yellow carotenoids give color for many flowers and fruits for attracting pollinators and seed dispersers. Carotenoids play also other essential roles in plants by being involved in photosystem assembly, light-harvesting and photoprotection [3]. Moreover, they serve as precursors for important carotenoid cleavage products called apocarotenoids, which include phytohormone abscisic acid (ABA), strigolactones and volatile flavor compounds [4–6]. Humans cannot biosynthesize carotenoids and, therefore, these essential compounds need to be acquired from the diet. Carotenoids are essential for humans as precursors of vitamin A but they also provide other health-benefits due to their antioxidant properties. Consumption of carotenoid rich food can enhance immune system and certain carotenoids have been shown to exert protective effects against cardiovascular diseases, certain types of cancers as well as degenerative diseases [7, 8]. Especially lutein and zeaxanthin have an ability to slow down age-related damage to the eye retina [9].

**Fig. 1:**
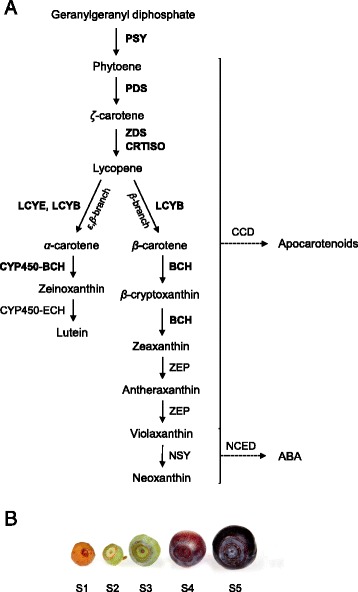
**a** The carotenoid biosynthetic pathway in higher plants. Modified according to [3, 10]. PSY, phytoene synthase; PDS, phytoene desaturase; ZDS, *ζ*-carotene desaturase; CRTISO, carotenoid isomerase; LCYB, lycopene *β*-cyclase; LCYE, lycopene *ε*-cyclase; BCH, *β*-carotene hydroxylase; CYP450-BCH, carotenoid *β*-ring hydroxylase of cytochrome P450 family; CYP450-ECH, carotenoid *ε*-ring hydroxylase of cytochrome P450 family; ZEP, zeaxanthin epoxidase; NSY, neoxanthin synthase; CCD, carotenoid cleavage dioxygenase; NCED, 9-*cis*-epoxycarotenoid dioxygenase. **b** Bilberry fruit developmental stages. S1, flower; S2, small unripe green fruit; S3, large unripe green fruit; S4, ripening purple fruit; S5, fully ripe blue fruit

Due to the importance of carotenoids to plants and humans, carotenoid biosynthetic pathway in plants (Fig. 1a) is well established and it takes place in plastids by nuclear-encoded enzymes [3, 6, 10]. The first committed step in the carotenoid biosynthesis, the condensation of two molecules of geranylgeranyl diphosphate (GGPP) to phytoene by phytoene synthase (PSY), has in many plant systems been reported as the rate-limiting step controlling the metabolic flux to carotenoid biosynthesis [3, 5]. By a series of reactions, phytoene is desaturated to lycopene involving the action of phytoene desaturase (PDS), *ζ*-carotene desaturase (ZDS), and at least two isomerases, including carotenoid isomerase (CRTISO) [3]. In some fruits, such as tomato (*Solanum lycopersicum* L.), the red lycopene is the major accumulating carotenoid compound. In the branching point of the carotenoid pathway, lycopene can be further cyclized by lycopene cyclases, lycopene *β*-cyclase (LCYB) and lycopene ε-cyclase (LCYE), to form either *α*-carotene or *β*-carotene. In the *ε*,*β*-branch, biosynthesis of lutein from *α*-carotene requires sequential action of two separate carotenoid hydroxylases belonging to the cytochrome P450 family, *β*-ring hydroxylase (CYP450-BCH) and *ε*-ring hydroxylase (CYP450-ECH) [10]. In the *β*-branch of the carotenoid pathway, hydroxylation of *β*-carotene by *β*-carotene hydroxylase (BCH) produces zeaxanthin via *β*-cryptoxanthin for the xanthophyll cycle. Epoxidation of the zeaxanthin in the ABA biosynthetic pathway leads to the formation of violaxanthin and neoxanthin, which can be further cleaved by 9-*cis*-epoxycarotenoid dioxygenase (NCED) to produce plant hormone ABA [3, 6, 11].

Previous studies on carotenoid biosynthesis have evidenced that in many fruits, the coordinated transcriptional regulation of the carotenoid biosynthetic genes, especially *PSY*, *PDS* and lycopene cyclases, is the key determinant of carotenoid profile [5, 12–15]. For example, tomato carotenoid composition coincides well with the up-regulation of the early carotenoid biosynthetic genes and the down-regulation of the downstream genes of the accumulating carotenoids [16]. However, beyond the transcript level, carotenoid metabolism is shown to be regulated in a much more complex manner by an elaborate coordinated regulatory network associated in fruit development and ripening [6]. Much of the knowledge of fruit carotenogenesis has been achieved from studies with tomato, a model system of the climacteric fruit ripening [5, 6], while the regulatory mechanisms affecting to the fruit ripening and carotenoid composition has been shown to vary in different fruit species and compared to the species showing non-climacteric fruit ripening [17]. In addition to the transcriptional regulation, factors such as post-transcriptional regulation, chromoplast biogenesis, epigenetic mechanisms and enzymatic degradation of carotenoids to apocarotenoids by the carotenoid cleavage dioxygenases (CCDs) can have a profound role on the fruit carotenoid metabolism [3, 5, 6]. In fact, some apocarotenoid compounds are recognized as important signaling, aroma, flavor and pigment components in fruits and berries [18–20].

In addition to the genetic factors, environmental factors, such as quantity of light, are recognized as regulators of carotenoid biosynthesis in chloroplasts. Although differential mechanisms are involved, there are few reports indicating the regulation of carotenogenesis in chromoplasts of fruits by light quality. In tomato and *Citrus* fruits, especially red light wavelengths have been demonstrated to increase carotenoid biosynthesis and accumulation [21, 22].

Bilberry (*Vaccinium myrtillus* L., Fig. 1b) is one of the most abundant wild berries in the Northern Europe recognized for its exceptionally high anthocyanin content indicated by the deep blue colour of ripe fruits [23]. Besides flavonoids, these berries are potential sources of other health-beneficial compounds and previously we have shown them to be a moderate source of vitamin C [24]. Although carotenoid biosynthesis has been studied in various fruit producing species, the reports of the carotenoid metabolism in non-climacteric *Vaccinium* fruits are scarce. Previous measurements have shown that lutein and *β*-carotene are the major carotenoids in ripe bilberry and blueberry (*V. corymbosum* L.) fruits [25–28] but studies on the carotenoid biosynthesis at different phases of fruit development and ripening are lacking. In the present work, we aimed to measure in detail the levels of carotenoids and analyze their biosynthesis and degradation during bilberry fruit development (Fig. 1b). For this purpose, eight carotenoid biosynthetic genes (*VmPSY*, *VmPDS*, *VmZDS*, *VmCRTISO*, *VmLCYB, VmLCYE, VmBCH* and *VmCYP450-BCH*) as well as a carotenoid cleavage gene *VmCCD1* were cloned, and their expression patterns were determined in the bilberry fruits at different stages of development and ripening. In addition, the effect of various light conditions on the carotenoid metabolism in unripe and mature berries was studied.

## Results

### Identification of carotenoid biosynthetic genes and *CCD1* in bilberry

In order to examine the bilberry fruit development related carotenoid biosynthesis at a molecular level, sequences of the genes phytoene synthase (*VmPSY*), phytoene desaturase (*VmPDS*), *ζ*-carotene desaturase (*VmZDS*), carotenoid isomerase (*VmCRTISO*), lycopene *β*-cyclase (*VmLCYB*), lycopene *ε*-cyclase (*VmLCYE*), *β*-carotene hydroxylase (*VmBCH*) and carotenoid *β*-ring hydroxylase of cytochrome P450 family (*VmCYP450-BCH*) were isolated. All the isolated sequences showed high identities to the corresponding sequences reported previously in the fruit carotenoid biosynthesis in other species (Table 1). Also the sequence of the isolated carotenoid cleavage gene *VmCCD1* of bilberry showed a high identity to the CCD1 class enzymes associated with carotenoid cleavage in other fruit species (Table 1). For example, *VmCCD1* is 89 % identical at the amino acid level to the *VvCCD1* of grape (*Vitis vinifera* L.) berry, which is implicated in the cleavage of zeaxanthin and lutein for the formation of C_13_- and C_14_-apocarotenoids [29]. All the obtained sequences from bilberry were deposited to the GenBank database (Table 1).

**Table 1:** The identity of carotenoid biosynthetic and cleavage genes of bilberry compared with other fruit species

Gene	GenBank accession no.	Clone size (bp)	Identity at amino acid level (%)
*VmPSY*	KR706538	806	93 (*Actinidia deliciosa*, ACO53104)
			92 (*Diospyros kaki*, ACM44688)
			89 (*Citrus sinensis*, ABB72444)
*VmPDS*	KR706539	828	92 (*Diospyros kaki*, ACY78343)
			91 (*Vitis vinifera*, AFP28796)
			90 (*Citrus sinensis*, ABB72445)
*VmZDS*	KR706540	553	86 (*Citrus sinensis*, NP_001275793)
			85 (*Vitis vinifera*, AFP28797)
			85 (*Lycium barbarum,* AIX87496)
*VmCRTISO*	KR706541	369	92 (*Lycium barbarum*, AIX87497)
			89 (*Solanum lycopersicum*, AAL91366)
			89 (*Citrus unshiu*, AIG20207)
*VmLCYB*	KR706542	487	93 (*Capsicum annuum*, ADH04271)
			93 (*Vitis vinifera*, AFP28799)
			91 (*Diospyros kaki*, ACR25158)
*VmLCYE*	KR706543	529	87 (*Diospyros kaki*, BAE94036)
			82 (*Coffea canephora*, ABC87738)
			81 (*Citrus limon*, BAD07293)
*VmBCH*	KR706544	783	78 (*Capsicum annuum*, CAA70888)
			77 (*Diospyros kaki*, ACN86365)
			76 (*Coffea arabica*, ABA43903)
*VmCYP450-BCH*	KR706545	715	82 (*Solanum lycopersicum* CYP97A29, ACJ25969)
			79 (*Lycium ruthenicum* CYP97A29, AIX87527)
*VmCCD1*	KR706546	909	89 (*Vitis vinifera*, AAX48772)
			87 (*Citrus sinensis*, BAE92958)
			86 (*Coffea arabica*, ABA43904)

### Expression of carotenoid biosynthetic genes and *CCD1* during bilberry fruit development

**Fig. 2:**
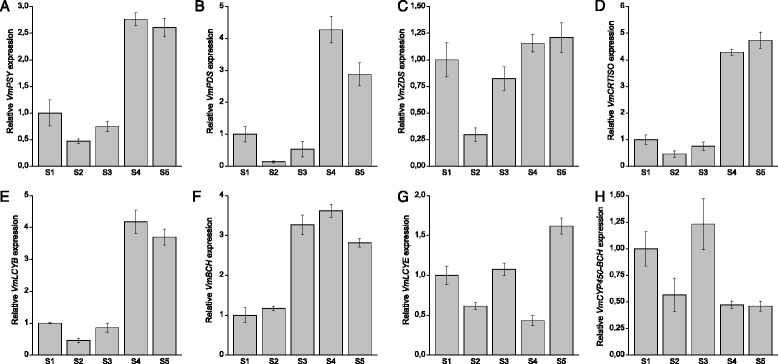
Expression of carotenoid biosynthetic genes *VmPSY* (**a**), *VmPDS* (**b**), *VmZDS* (**c**), *VmCRTISO* (**d**), *VmLCYB* (**e**), *VmBCH* (**f**), *VmLCYE* (**g**) and *VmCYP450-BCH* (**h**) during bilberry fruit development. The relative expression of the genes was quantified by qRT-PCR and normalized to *VmGAPDH*. Values represent means ± SEs of at least three replicates. S1–S5 indicates the fruit developmental stages from flower to fully ripe berry

**Fig. 3:**
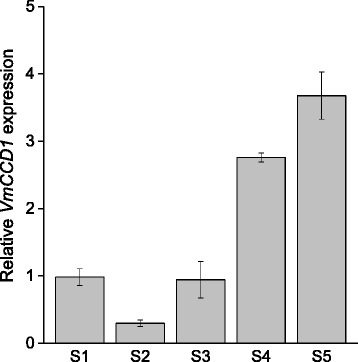
Expression of *VmCCD1* gene during bilberry fruit development. The relative expression of the gene was quantified by qRT-PCR and normalized to *VmGAPDH*. Values represent means ± SEs of at least three replicates. S1–S5 indicates the fruit developmental stages from flower to fully ripe berry

Expression of the carotenoid biosynthetic genes and the cleavage gene *VmCCD1* was analyzed in bilberry fruit at five different developmental stages (Fig. 1b) by qRT-PCR. All the eight examined biosynthetic genes were expressed at detectable levels throughout bilberry fruit development but with variable expression patterns (Fig. 2). The expression of the early biosynthetic genes *VmPSY*, *VmPDS* and *VmCRTISO* showed highly similar patterns with relatively low expression at the early stages of fruit development but approximately four-, eight- and six-fold increment, respectively, at the onset of fruit ripening (S4). Their expression was relatively high also in ripe fruit (S5). The expression of *VmZDS* was high in flowers (S1) decreasing at the beginning of fruit development and increasing again towards the ripe fruit. The expression of *VmLCYB,* which has a role both in *ε*,*β*- and *β*-branches of the carotenoid pathway, resembled that of *VmPSY*, *VmPDS* and *VmCRTISO* showing a five-fold increment in its expression at the fruit ripening (S4) while the expression of *VmBCH,* a *β*-branch gene, showed increase already at the green fruit stage (S3). The transcript levels of the genes specific to *ε*,*β*-branch, *VmLCYE* and *VmCYP450-BCH*, were relatively high at the stage of flowering (S1) as well as at the green stage (S3) but were down-regulated at the onset of fruit ripening (S4). The expression of the *VmLCYE* was up-regulated again in ripe berries (S5). The expression of the carotenoid cleavage gene *VmCCD1* was found to increase during the berry development (Fig. 3), especially at the stage of fruit ripening (S4), resembling the expression patterns of *VmPSY*, *VmPDS*, *VmCRTISO* and *VmLCYB.*

### Carotenoid content during bilberry fruit development

**Fig. 4:**
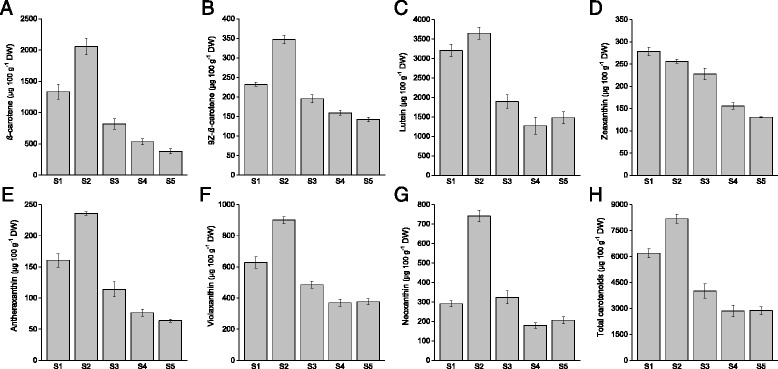
Content of *β*-carotene (**a**), 9Z-*β*-carotene (**b**), lutein (**c**), zeaxanthin (**d**), antheraxanthin (**e**), violaxanthin (**f**)*,* neoxanthin (**g**), and total carotenoids (**h**) during bilberry fruit development. Values represent means ± SEs of four replicates. S1–S5 indicates the fruit developmental stages from flower to fully ripe berry. DW, dry weight

The carotenoid composition and concentrations in the bilberry fruit were analyzed in detail at the five developmental stages (Fig. 1b) by HPLC-DAD revealing the presence of carotenes and xanthophylls. Among carotenes, *β*-carotene and smaller amount of 9Z-*β*-carotene were detected whereas xanthophylls detected in bilberry fruit included lutein, zeaxanthin, antheraxanthin, violaxanthin, neoxanthin (Fig. 4) and traces amounts of *β*-cryptoxanthin. No lycopene was detected in bilberry fruit.

Lutein was found to be the most abundant carotenoid in bilberry fruit at every developmental stage followed by *β*-carotene (Fig. 4). The levels of both lutein and *β*-carotene were highest in small unripe green fruit (S2) and the amounts decreased during the fruit development although there was a slight increase in the lutein concentration at the end of fruit ripening. In ripe fruit, the final concentrations of lutein and *β*-carotene were 1476 μg 100 g^−1^DW (184 μg 100 g^−1^ FW) and 380 μg 100 g^−1^DW (47 μg 100 g^−1^ FW), respectively. Also the levels of 9Z-*β*-carotene, zeaxanthin, antheraxanthin, violaxanthin and neoxanthin showed a decreasing trend during the bilberry fruit development (Fig. 4). The highest levels of these compounds were detected in small unripe green fruit (S2) with the exception of zeaxanthin concentration being the highest in flowers (S1). The total carotenoid content in the ripe bilberry fruit was 2872 μg 100 g^−1^DW (359 μg 100 g^−1^ FW) of which approximately 64 % constituted of lutein and *β*-carotene.

### Expression of carotenoid biosynthetic genes in bilberry fruit at different light conditions

**Fig. 5:**
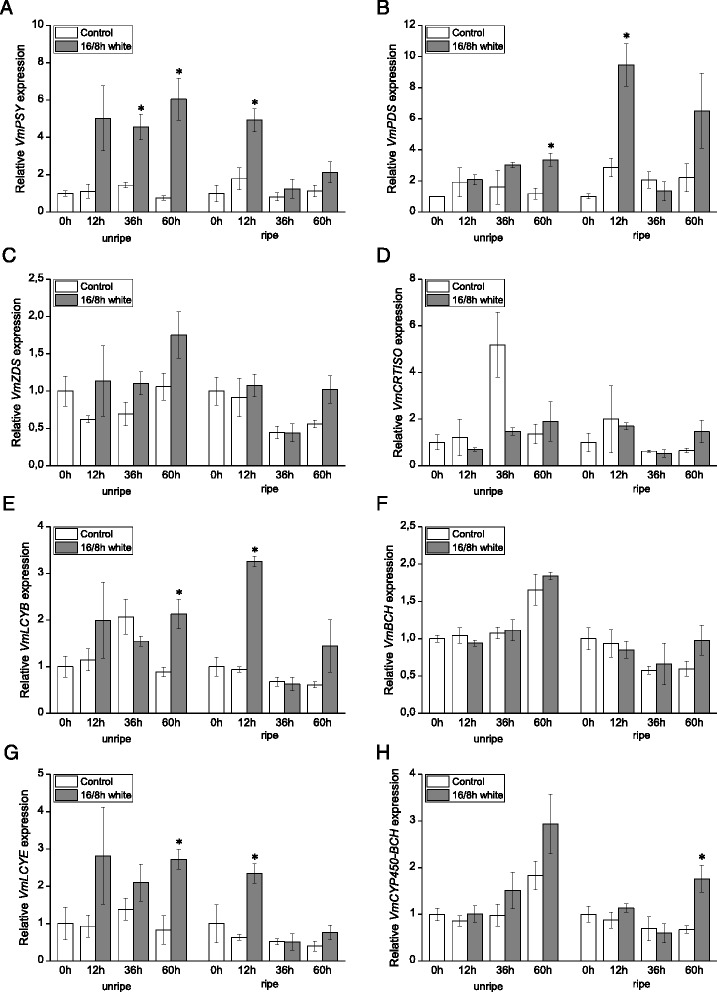
Effect of 16/8 h photoperiodic white light treatment on the expression of carotenoid biosynthetic genes *VmPSY* (**a**), *VmPDS* (**b**), *VmZDS* (**c**), *VmCRTISO* (**d**), *VmLCYB* (**e**), *VmBCH* (**f**), *VmLCYE* (**g**) and *VmCYP450-BCH* (**h**) in unripe (stage S3) and ripe (stage S5) bilberry fruits. The relative expression of the genes was quantified by qRT-PCR and normalized to *VmGAPDH*. Values represent means ± SEs of three replicates. Asterisks indicate statistically significant differences from respective control (dark treatment) in Student’s *t*-Test (*P* ≤ 0.05)

Expression of the carotenoid biosynthetic genes was evaluated after treatment of unripe (S3) and ripe (S5) bilberry fruits with white light. In both unripe and ripe berries, photoperiodic (16/8 h) white light significantly induced the expression of *VmPSY*, *VmPDS*, *VmLCYB* and *VmLCYE* compared to the control berries kept in darkness (Fig. 5). The expression of the other biosynthetic genes, *VmZDS*, *VmCRTISO*, *VmBCH* and *VmCYP450-BCH*, did not show a marked response to the photoperiodic white light treatment, although the increase in the *VmCYP450-BCH* expression in ripe fruit after 60 h treatment was significant.

**Fig. 6:**
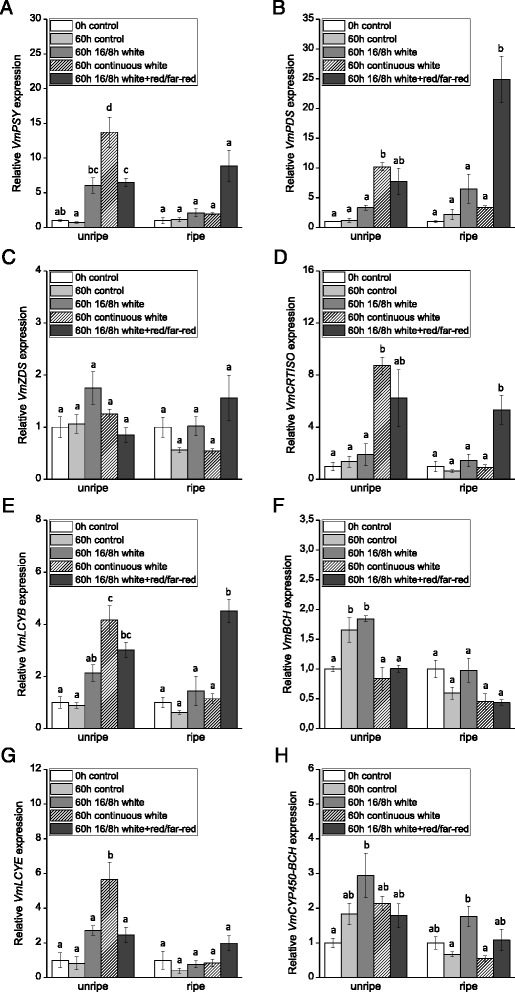
Effect of different light conditions on the expression of carotenoid biosynthetic genes *VmPSY* (**a**), *VmPDS* (**b**), *VmZDS* (**c**), *VmCRTISO* (**d**), *VmLCYB* (**e**), *VmBCH* (**f**), *VmLCYE* (**g**) and *VmCYP450-BCH* (**h**) in unripe (stage S3) and ripe (stage S5) bilberry fruits. The relative expression of the genes was quantified by qRT-PCR and normalized to *VmGAPDH*. Values represent means ± SEs of three replicates. Columns labeled with different letters indicate statistically significant differences (*P* ≤ 0.05, one-way ANOVA with *post hoc* comparisons)

The effect of photoperiod and light quality on bilberry fruit carotenoid biosynthesis was further investigated by a 60 h-treatment with continuous (24 h) white light and photoperiodic (16/8 h) white light with elevated red/far-red wavelengths. In unripe berries, the treatment with continuous white light had a significant increasing effect on the expression of *VmPSY*, *VmPDS*, *VmCRTISO*, *VmLCYB* and *VmLCYE* compared with the control berries kept in darkness as well as the berries grown under photoperiodic white light (Fig. 6). Instead, the expression of *VmZDS*, *VmBCH* and *VmCYP450-BCH* in unripe berries was not elevated by the continuous white light treatment compared to the photoperiodic white light treatment. The additional red/far-red light wavelengths did not have a significant elevating effect on the gene expression in unripe berries compared to the photoperiodic white light treatment, although a slight increase in the expression of *VmPDS*, *VmCRTISO* and *VmLCYB* was observed. In ripe berries, light had differential effect on the expression of the carotenoid biosynthetic genes compared to unripe berries and, generally, continuous and photoperiodic white light treatments had no or only a slight inducing effect on the expression of the carotenoid biosynthetic genes after 60 h treatment (Fig. 6). Instead, the expression of *VmPSY*, *VmPDS*, *VmCRTISO*, *VmLCYB* and *VmLCYE* was increased in ripe berries grown under elevated red/far-red light wavelengths in the 16/8 h photoperiod compared to all the other treatments. The expression of *VmZDS*, *VmBCH* and *VmCYP450-BCH* in ripe berries did not show a marked response to the treatment.

### Expression of carotenoid cleavage genes in bilberry fruit at different light conditions

**Fig. 7:**
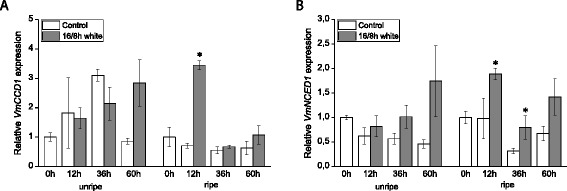
Effect of 16/8 h photoperiodic white light treatment on the expression of carotenoid cleavage genes *VmCCD1* (**a**) and *VmNCED1* (**b**) in unripe (stage S3) and ripe (stage S5) bilberry fruits. The relative expression of the genes was quantified by qRT-PCR and normalized to *VmGAPDH*. Values represent means ± SEs of three replicates. Asterisks indicate statistically significant differences from respective control (dark treatment) in Student’s *t*-Test (*P* ≤ 0.05)

**Fig. 8:**
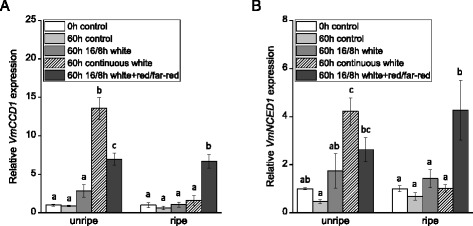
Effect of different light conditions on the expression of carotenoid cleavage genes *VmCCD1* (**a**) and *VmNCED1* (**b**) in unripe (stage S3) and ripe (stage S5) bilberry fruits. The relative expression of the genes was quantified by qRT-PCR and normalized to *VmGAPDH*. Values represent means ± SEs of three replicates. Columns labeled with different letters indicate statistically significant differences (*P* ≤ 0.05, one-way ANOVA with *post hoc* comparisons)

Expression of the carotenoid cleavage genes, *VmCCD1* and the previously isolated *VmNCED1*, the key gene in bilberry ABA biosynthesis [11], was measured after the treatment of unripe and ripe bilberry fruits with different light conditions. The results show that the expression of both of these cleavage genes could be induced by a 16/8 h photoperiodic white light treatment (Fig. 7). Continuous white light exposure for 60 h had a significant inducing effect on the expression of the cleavage genes in unripe berries compared with the control berries kept in darkness as well as the berries grown under photoperiodic white light (Fig. 8). Elevation of red/far-red light wavelengths in photoperiodic white light had a significant increasing effect on the expression of *VmCCD1* and it also slightly increased the expression of *VmNCED1* in unripe berries. In ripe berries, the 60 h white light treatments did not significantly affect the gene expression but instead red/far-red light wavelengths had a significant inducing effect on the expression of both of the cleavage genes.

### Carotenoid content of bilberry fruit at different light conditions

The effect of different light conditions on the accumulation of carotenoids was analyzed after five days of the initiation of each light treatment. The metabolic profile of unripe berries showed a slight increment in the accumulation of both *β*-branch and *ε*,*β*-branch carotenoids after white light exposure (Table 2). The increase in *β*-carotene concentration was significant after five days treatment with continuous white light compared to the dark grown control berries. On the contrary, in ripe berries, all the light treatments led to the decreased content of carotenoids compared to the berries grown in dark (Table 2).

**Table 2:** Effect of light conditions on carotenoid content (μg 100 g ^−1^ DW) in bilberry fruits

Compound	Light treatment for unripe fruits				Light treatment for ripe fruits			
	Control	16/8 h white	Continuous white	16/8 h white + red/far-red	Control	16/8 h white	Continuous white	16/8 h white + red/far-red
Lutein	939.44 ± 95.84	1170.85 ± 453.17	1221.14 ± 119.55	892.26 ± 233.49	969.27 ± 324.59	457.25 ± 21.71	564.34 ± 61.60	503.88 ± 75.53
*β*-carotene	296.52 ± 16.48 a	392.43 ± 137.69 ab	469.43 ± 15.71 cb	265.46 ± 90.87 ab	342.62 ± 53.00	294.33 ± 8.92	251.40 ± 12.38	302.11 ± 15.34
9Z-*β*-carotene	140.30 ± 6.57	158.06 ± 36.20	159.82 ± 2.90	141.29 ± 15.52	148.86 ± 8.42	140.74 ± 2.74	141.51 ± 4.22	141.90 ± 2.27
Zeaxanthin	ud	162.76 ± 16.74	185.18 ± 5.22	187.94 ± 18.35	ud	ud	ud	ud
Antheraxanthin	67.37 ± 5.35	84.91 ± 15.01	69.15 ± 0.85	70.55 ± 8.23	69.29 ± 15.33	ud	54.65 ± 1.22	ud
Violaxanthin	356.08 ± 29.29	409.93 ± 115.15	ud	291.98 ± 13.36	425.91 ± 62.40	320.89 ± 17.33	324.18 ± 24.68	306.41 ± 4.82
Neoxanthin	272.28 ± 51.13	356.33 ± 33.22	310.67 ± 67.01	301.75 ± 67.64	228.60 ± 41.17	154.95 ± 4.05	187.90 ± 5.48	179.80 ± 11.63
*β* -cryptoxanthin	ud	ud	ud	ud	ud	ud	128.00 ± 90.51	132.33 ± 76.40
Total carotenoids	2071.99 ± 140.58	2681.03 ± 703.42	2508.56 ± 241.57	1979.33 ± 547.75	2163.68 ± 485.14	1368.16 ± 46.20	1505.76 ± 82.74	1451.98 ± 107.76

Values represent means ± SEs of three replicates

Only statistically significant differences are presented by different letters (*P* ≤ 0.05, one-way ANOVA with *post hoc* comparisons)

*DW* dry weight, *ud* under detection limit

## Discussion

### Bilberries are good source of carotenoids among fruits and berries

Carotenoids have been shown to accumulate in chromoplasts of fruits with variable profiles and concentrations between species and even between close cultivars [30–33]. Bilberry fruits accumulate high amounts of anthocyanin pigments during the ripening. The biosynthesis and content of carotenoids during the fruit development and ripening has not been evaluated earlier in the genus *Vaccinium*. The results of the current study are in agreement with the earlier measurements that have indicated lutein and *β*-carotene as the main carotenoids in ripe bilberry fruits [25–28]. Our study demonstrates that the total carotenoid content in ripe bilberries is higher than described for example in ripe fruits of raspberry (*Rubus idaeus* L.), grape berry, strawberry (*Fragaria* × *ananassa*) or commercial apple (*Malus* × *domestica*) cultivars [25, 31, 34, 35] indicating that among fruits and berries, bilberries can be considered as a good source of carotenoids. Therefore, carotenoids contribute to the overall antioxidant capacity of bilberry fruit in addition to the high anthocyanin [23] and modest ascorbic acid [24] contents.

### Carotenoid content during bilberry fruit development is determined by different molecular mechanisms

In some fruits, such as tomato, *Citrus*, red-fleshed watermelon (*Citrullus lanatus*) and sea buckthorn (*Hippophae rhamnoides* L.), the carotenoid content increases during the fruit maturation indicated by the appearance of yellow to red color in ripening fruit [14, 33, 36, 37]. In other fruits, such as strawberry, raspberries, grape and apple, in which the red pigment formation is mostly a consequence of anthocyanin accumulation, a decreasing trend in the carotenoid content over the fruit development has been described [18, 19, 31, 34, 35]. According to the current results, bilberry belongs to the latter group showing a decreasing trend in the content of both carotenes and xanthophylls during the fruit development. The detected decrease in the levels of all carotenoid compounds over the fruit development does not coincide with the notable increment in the expression of the carotenoid biosynthetic genes at the fruit ripening demonstrated in our current study. The up-regulation of the early biosynthetic genes (*VmPSY*, *VmPDS, VmZDS, VmCRTISO*), which generate the flux to carotenoids, as well as the up-regulation of the *VmLCYB* with simultaneous down-regulation of the specific genes of the *ε*,*β*-branch (*VmLCYE* and *VmCYP450-BCH)* at the ripening stage, indicates direction of the carotenoid biosynthesis towards *β*-branch at fruit ripening. However, none of the carotenoid compounds in the *β*-branch accumulates in the ripening bilberries. This suggests mechanisms beyond transcriptional regulation in the bilberry fruit carotenoid metabolism, and turnover of the carotenoid compounds from the pathway. Although the balance in the expression of the early and late biosynthetic genes is described as a key determinant of the carotenoid profile in many fruits [12–16], also other factors such as post-transcriptional mechanisms and enzymatic degradation of carotenoids to apocarotenoids by CCDs can affect to the final carotenoid content of fruits [3, 6]. Moreover, xanthophylls can also be found esterified with fatty acids as in the case of lutein esters reported in raspberries [35].

### Role of enzymatic degradation in carotenoid content during bilberry fruit ripening

The CCD1s are cytosolic localized enzymes that have a role in the cleavage of double bonds of carotenoids to form C_13_- and C_14_-apocarotenoids. These enzymes have multiple substrates, including C_27_-carotenoids in cytosol and C_40_-carotenoids accessed by CCD1 in the outer plastid envelope [38, 39]. In the present study, *VmCCD1* showing a high identity to other fruit *CCD1* genes was isolated from bilberry fruit and its expression was found to be up-regulated at the onset of bilberry fruit ripening. The high *CCD1* expression upon fruit development has earlier been associated with the carotenoid degradation and the formation of apocarotenoids in different fruit species [31, 40–42]. In raspberries, the decrease in the carotenoid content with the parallel increase in the *RiCCD1* expression during fruit ripening was suggested to be associated with the exceptionally high accumulation of apocarotenoid aroma volatiles in ripe fruit [18]. Also in grape berries approaching ripening, the increased expression of the *VvCCD1,* cleaving a variety of carotenoid substrates, led to the increased C_13_-norisoprenoid level in the ripe berries of Muscat of Alexandria and Shiraz cultivars [29, 43]. On the other hand, in strawberry, the up-regulation of *FaCCD1* expression at fruit ripening was suggested to be related with the simultaneous decrease in the carotenoid content, especially lutein [19]. Therefore, in the light of the previous studies concerning CCD1 function in fruits, the detected up-regulation of the *VmCCD1* in the ripening bilberries, may have a role in decreasing the carotenoid content towards ripe fruit, especially in the *β*-branch of the carotenoid pathway.

In our earlier study, we have shown that another well-known CCD member, *VmNCED1,* which encodes the key enzyme in the formation of ABA, shows an increase in its expression in the developing bilberry fruit leading to an elevated ABA level at the onset of fruit ripening [11]. This is a similar observation with other non-climacteric fruits, including strawberry and blueberry (*V. corymbosum*), in which ABA accumulation is considered as an initiator of fruit ripening and anthocyanin production [44, 45]. The suppression of *NCED1* expression blocking the metabolic flux to ABA has been shown to lead to an increased accumulation of upstream carotenoid compounds in tomato [37]. It is possible that the elevated *VmNCED1* expression at fruit ripening [11], which increases the degradation of violaxanthin and neoxanthin, can affect the carotenoid composition in ripening bilberry fruit. Since anthocyanins are responsible for the pigmentation of ripe bilberry fruits, the transition from photosynthetic green fruit to non-photosynthetic ripening fruit can readily involve degradation and reuse of the carotenoids for the formation of apocarotenoid compounds, such as ABA and volatile aroma compounds, which have been reported in ripe bilberries [11, 46]. However, other yet unidentified CCD family genes may also be involved in the carotenoid degradation in bilberry.

### Light has differential effect on the carotenoid metabolism between unripe and ripe berries

In addition to the programmed developmental regulation, light conditions seem to influence carotenoid biosynthesis in fruit tissues [21]. The results of the current study show that light up-regulates the expression of the carotenoid pathway genes in the bilberry fruit. The expression of particularly *VmPSY*, *VmPDS*, *VmCRTISO*, and both lycopene cyclases was stimulated by light. Despite of the significant increase in the transcript abundance of the carotenoid biosynthetic genes by all tested 60 h light treatments, only a slight increase in the carotenoid content in the unripe bilberry fruits was detected after white light treatments in the current study. In ripe berries, the carotenoid content decreased after the light treatments although red wavelengths were shown to increase the expression of the biosynthetic genes (Table 2). This inconsistency between the expression of the biosynthetic genes and the carotenoid content may be attributed to the post-transcriptional regulation or carotenoid degradation. Our current and previous studies [11] suggest the carotenoid degradation to be involved. First, as discussed earlier, the expression of the both *VmCCD1* and *VmNCED1* are higher in ripening bilberry fruits compared to the unripe fruits possibly explaining the decrease in the carotenoid content in the ripe fruit. Secondly, the expression of the both *VmCCD1* and *VmNCED1* were found in this study also to be up-regulated by the light treatments, which could lead to a higher rate of carotenoid breakdown. The up-regulation of the *NCED1* expression by light has been reported earlier in the ripening grape berry [47, 48] as well as in *Citrus* where elevated expression of the *NCED* genes by red light treatment was demonstrated in pulp tissues of three different citrus varieties [49]. Short treatments with white, red or red/far-red light were demonstrated to up-regulate *PhCCD1* expression also in dark-adapted *Petunia* flowers by a phytochrome-independent manner, leading to a high volatile emission [50]. Considering bilberry fruits, our results indicate that despite of the higher flux of metabolites directed to the carotenoid pathway under high light conditions, the carotenoid content decreases due to the increment in the carotenoid cleavage reactions.

Some earlier reports have indicated that light quality and especially red light wavelengths affect the carotenoid biosynthesis in fruits. In post-harvest studies with tomato, red light treatments were demonstrated to increase lycopene content of fruit [21, 51]. In *Citrus*, the effect of red light treatment on the carotenoid biosynthesis and content was cultivar dependent [22, 49]. Our results showing the up-regulation of the carotenoid biosynthesis as a response to the red light are in agreement with these studies. Interestingly, we found the effect of the light quality on the expression of both the carotenoid biosynthetic and the cleavage genes to be dependent on fruit developmental stage. In the unripe berries, all the applied 60 h light treatments induced the expression of the carotenoid biosynthetic and cleavage genes while only red/far-red light wavelengths showed inducing effect in the ripe fruits. The detected differential effect of the light quality on the carotenoid metabolism between the developmental stages can be associated with the differences in the regulation of carotenoid biosynthesis between chloroplasts of unripe fruit and chromoplasts of ripe fruit. Unlike chloroplasts, the carotenoid content and composition in chromoplasts has been shown to vary highly between species and cultivars. It is possible that the red light has a specific role in the metabolism of fruits close to maturity. During tomato fruit ripening, the amount of red light passing through the pericarp tissues was shown to increase four-fold [51]. Therefore, the detected differential effect of the light quality can also be a result of the absorption of certain wavelengths of light by pigments present in the skin of the ripe fruits, such as anthocyanins in the ripe bilberries. Supporting this assumption, it has been shown that anthocyanins strongly absorb wavelengths under 600 nm in the leaf tissues [52]. The specific role of the red light on the ripening berry tissues and the carotenoid metabolism is supported by the study of Kondo et al. [53] in the grape berries. They showed that especially red light given to the ripening berries induced the expression of the *VvNCED1* and the formation of the plant hormone ABA also leading to the higher anthocyanin concentration. In the non-climacteric grape berries ABA is considered, similarly to *Vaccinium* berries, as an initiator of the ripening and the anthocyanin biosynthesis.

## Conclusions

This is the first report regarding to the carotenoid metabolism and its regulation in the berries of the genus *Vaccinium,* which are known for the high anthocyanin and antioxidant activity levels. The results show that among fruits and berries, bilberries are relatively good source of carotenoids, which also contribute to the overall antioxidant activity of the berries. An inconsistency between the carotenoid content and the related biosynthetic gene expression was detected during the bilberry fruit development, indicating a cleavage of carotenoids to apocarotenoids toward ripening of the berries. As a non-climacteric fruit, increase in the ABA formation at the onset of the fruit ripening can contribute to the rapid carotenoid turnover in bilberry. Moreover, light was shown to up-regulate both the carotenoid biosynthetic and the cleavage genes but only red/far-red light wavelengths had a role in the ripe bilberry fruits. Our results indicate that in the ripening bilberries, the red light wavelengths specifically induce the expression of the carotenoid metabolism genes directed to the apocarotenoid formation. Whether this has any effect on the flavor properties of bilberries, requires further investigations. It is also to be studied whether the red light wavelengths can induce the expression of the flavonoid biosynthetic genes in the mature *Vaccinium* berries through the ABA regulation.

## Methods

### Plant material and treatments

All plant material used for the experiments was originated from the natural forest stand in Oulu (65°01’ N, 25°28’ E), Finland. All material sampling was conducted complying with national guidelines. Five developmental stages of bilberry (*Vaccinium myrtillus* L.) fruit (Fig. 1b) were collected from June to August 2011. The developmental stages of the fruits were S1, flower collected June 7^th^ (anthesis); S2, small unripe green fruit (15 days after anthesis); S3, large unripe green fruit just before coloring began (28 days after anthesis); S4, ripening purple fruit (34 days after anthesis) and S5, fully ripe blue fruit (55 days after anthesis) as described earlier [11]. Immediately after collection, all samples were frozen in liquid nitrogen and stored at −80 °C until they were used for RNA extraction and determination of carotenoids.

In order to study the effect of the light conditions on the carotenoid metabolism, different light treatments were applied to the bilberry plants holding fruits: photoperiodic 16/8 h (light/dark) white light, continuous white light (24 h) and photoperiodic 16/8 h (light/dark) white light enhanced with red and far-red light wavelengths. Control plants were kept in the constant darkness. Light treatments were applied to the three replicate collections of bilberries at two different stages of fruit development; either on unripe S3 stage (collected at June 13^th^, 2013) or mature S5 stage (collected at July 3^rd^, 2013). In each separate experiment, bilberries were initially kept in the darkness for 24 h before exposed for five days to the different light treatments. During the light treatments, bilberries were placed under VP3411 M16 -light emitting diode (LED) lamps (Valopaa, Oulu, Finland) and irradiated with continuous or photoperiodic white light (400–800 nm; Additional file 1). The additional red to far-red light (600–800 nm) was provided combining the VP3411 M16 -LED lamps with Ecoline R7s halogen lamps (Osram, Vantaa, Finland) filtrated by the Lucite Abmer 300 transparent plex (Additional file 1). The light intensity, measured by the USB RAD+ spectroradiometer (Ocean Optics Inc., Dunedin, FL, USA), was 152 μmol m^−2^ s^−1^ under the white light treatment and 200 μmol m^−2^ s^−1^ under the white + red/far-red light treatment. The increase in the light intensity in white + red/far-red light treatment was solely due to the increase in the red to far-red light wavelengths (Additional file 1). In the white light treatments, the red/far-red light ratio was 8.9 and after the supplementation of the white light with red to far-red light the ratio was 1.5. Temperature in the growth room was maintained 20–21 °C throughout the experiments. Berry samples were collected after 0, 12, 36 and 60 h from the beginning of the light treatments for the gene expression analyses and at day 5 (116 h) for the analyses of carotenoids. Immediately after the excision, all berry samples were frozen in liquid nitrogen and stored at −80 °C until they were used for RNA extraction and determination of carotenoids. The plant material was identified by Dr. Marian Sarala and voucher samples are stored at the Genetics and Physiology Unit, University of Oulu, Finland.

### Isolation of RNA and cDNA preparation

Total RNA was isolated from the samples according to the method described for bilberry [54]. The cDNA was synthesized from the total RNA using SuperScript III reverse transcriptase (Invitrogen, Carlsbad, CA, USA) according to the manufacturer’s instructions. The cDNA was purified from the contaminating genomic DNA by using the method described by Jaakola et al. [55].

### Isolation of carotenoid biosynthetic genes

The amplification of the sequences of the key carotenoid biosynthetic genes, phytoene synthase (*VmPSY*), phytoene desaturase (*VmPDS*), *ζ*-carotene desaturase (*VmZDS*), carotenoid isomerase (*VmCRTISO*), lycopene *β*-cyclase (*VmLCYB*), lycopene *ε*-cyclase (*VmLCYE*), *β*-carotene hydroxylase (*VmBCH*) and carotenoid *β*-ring hydroxylase of cytochrome P450 family (*VmCYP450-BCH*) as well as the carotenoid cleavage dioxygenase class 1 (*VmCCD1*), was achieved from berry cDNA with gene-specific primers that were designed based on the sequences of the respective genes found in *V. corymbosum* transcriptome database [56]. The PCR-reactions were performed by using DyNazyme™ II DNA polymerase (Finnzymes, Espoo, Finland). The amplified PCR products were gel-purified using Montage® DNA Gel Extraction Kit (Millipore, Bedford, MA, USA). The purified PCR products were ligated into a pGEM-T Easy vector (Promega, Madison, WI, USA) and sequenced using an ABI 3730 DNA sequencer (Applied Biosystems, Foster City, CA, USA) with a BigDye Terminator Cycle Sequencing Kit (Applied Biosystems).

### Relative quantification of gene expression

Real-time quantitative reverse transcription PCR (qRT-PCR) analyses were performed with a LightCycler 480 instrument and software (Roche Applied Sciences, Indianapolis, IN, USA). The transcript abundance of the bilberry carotenoid biosynthetic genes (*VmPSY*, *VmPDS*, *VmZDS*, *VmCRTISO*, *VmLCYB*, *VmLCYE*, *VmBCH*, *VmCYP450-BCH*) and the cleavage genes (*VmCCD1*, *VmNCED1;* GenBank accession no. JX982599) was detected using a LightCycler® SYBR Green I Master qPCR kit (Roche). The qRT-PCR conditions were an initial incubation at 95 °C for 10 min followed by 45 cycles of 95 °C for 10 s, 60 °C for 20 s, and 72 °C for 10 s. The gene-specific primer sequences used for the qRT-PCR analysis are shown in Table 3. Glyceraldehyde-3-phosphate dehydrogenase (*VmGAPDH*; GenBank accession no. AY123769) was used as a reference gene for the relative quantification of the PCR products. The results were calculated with LightCycler® 480 software (Roche), using the calibrator-normalized PCR efficiency-corrected method (Technical note no. LC 13/2001, Roche). The amplification of only one product in qRT-PCR was confirmed by a melting curve analysis and sequencing.

**Table 3:** Gene-specific primers used for qRT-PCR analysis

Gene	Primer sequence 5’-3’
*VmPSY*	ATGCATCGCACATAACTCCA (forward)
	GGTCCATCCTCATTCCTTCA (reverse)
*VmPDS*	GTTGCAGCGGAAAGAACATT (forward)
	CATTGCTGGCAGTAGTCCAA (reverse)
*VmZDS*	TGCCATTACCAAATGACGAA (forward)
	ATATGAGCCAGCGAGGAAGA (reverse)
*VmCRTISO*	TCTTGAGTGCTTGACGCTTG (forward)
	GGGTAGTTGATTCCCCCAAA (reverse)
*VmLCYB*	ACGGCGTTAAGTTCCATCAA (forward)
	TCAGCCAAAATCCCATAAGC (reverse)
*VmLCYE*	TCGTTCTTACGGGCGAGTTA (forward)
	TTCCAGAAGCTGCTCCAGAT (reverse)
*VmBCH*	TATCGGAGATGTTTGGCACA (forward)
	ACCGCGTTTATAATGGCAAA (reverse)
*VmCYP450-BCH*	ATGTGTTGGCGACATGTTTG (forward)
	GCATCTCCAATTCAGGCAGT (reverse)
*VmCCD1*	ATGCTGAGAGCAAGGCTGAAAG (forward)
	ATCCAACATGCCAAGAGTCTGC (reverse)
*VmNCED1*	CCCGAACAGGGGAGGATATT (forward)
	CGGTCAACACGGACTTCAAA (reverse)
*VmGAPDH*	CAAACTGTCTTGCCCCACTT (forward)
	CAGGCAACACCTTACCAACA (reverse)

### Analysis of carotenoids

Carotenoids were extracted using the method described earlier [35] with small modifications. Freeze-dried flowers and berries were ground into a fine powder under liquid nitrogen using a mortar and pestle. Fifty milligrams of each sample was mixed with 6 % methanolic KOH, vortexed vigorously and incubated for 2.5 h at room temperature. Carotenoids were extracted twice with 1 ml of hexane and the extracts were dried by centrifugal evaporation. The dried residues were stored at −20 °C before dissolved in 50 μl ethyl-acetate for HPLC. All steps were carried out, when possible, on ice and shielded from light to prevent carotenoid degradation.

HPLC-analysis was performed according to Fraser et al. [57] by using a 1290 Agilent UPLC equipped with a reverse-phase C_30_ column (250 × 2.1 mm) with 3 μm particle size coupled to a C_30_ 20 × 4.6 mm guard column (YMC Inc., Wilmington, NC, USA). The mobile phases consisted of methanol (A) and tert-methyl butyl ether containing 5 % of water/methanol (20/80 v/v) and 0.2 % (w/v) ammonium acetate (B). The gradient elution used was 100 % A, 0 % B isocratically for the initial 6 min, a step to 82.5 % A and 17.5 % B at 7 min maintained for 5 min, followed by a linear gradient to 32.5 % A and 67.5 % B by 32 min, which was maintained for 14 min. A conditioning phase (48–60 min) was used to return the column to the initial concentrations of A and B. Flow rates of 0.21 ml min^−1^ were used and injection volume was 3 μl. The DAD (diode array detector) signal was acquired monitoring the eluate continuously from 200 nm to 600 nm. Carotenes and xanthophylls were quantified considering the area of each peak at a wavelength of 450 nm. Quantification of individual compounds was performed by external standard calibration curves by using the respective standards and the script developed by Wehrens et al. [58].

### Statistical analysis

Quantitative results of the gene expression and metabolite analyses are presented in terms of means ± SEs of at least three biological replicates. The effect of light conditions on the gene expression and the carotenoid content were analysed by either Student’s *t*-Test or one-way analysis of variance (ANOVA) followed by Tukey’s HSD test (or in cases where the homogeneity of variances assumption was not met, the Games-Howell test) using SPSS Statistics program, version 22 (IBM, New York, NY, USA).

### Ethics and consent to participate

Not applicable.

### Consent to publish

Not applicable.

### Availability of data and materials

The data sets supporting the results of this article are included within the article and its additional files. All the obtained sequences from bilberry were deposited to the GenBank database and the accession numbers are shown in Table 1.

## Additional file

Additional file 1: Spectra of light treatments applied to bilberry fruits. (PDF 27 kb)

## Abbreviations

ABA: abscisic acid
BCH: *β*-carotene hydroxylase
CCD: carotenoid cleavage dioxygenase
CRTISO: carotenoid isomerase
CYP450-BCH: carotenoid *β*-ring hydroxylase of cytochrome P450 family
LCYB: lycopene *β*-cyclase
LCYE: lycopene *ε*-cyclase
NCED: 9-*cis*-epoxycarotenoid dioxygenase
PDS: phytoene desaturase
PSY: phytoene synthase
qRT-PCR: real-time quantitative reverse transcription PCR
ZDS: *ζ*-carotene desaturase
