# Supplementary figures and images for: Carotenoid metabolism during bilberry (Vaccinium myrtillus L.) fruit development under different light conditions is regulated by biosynthesis and degradation
Source: BMC Plant Biol. 2016 Apr 21;16:95. doi: 10.1186/s12870-016-0785-5 (PMC4839083; doi:10.1186/s12870-016-0785-5)

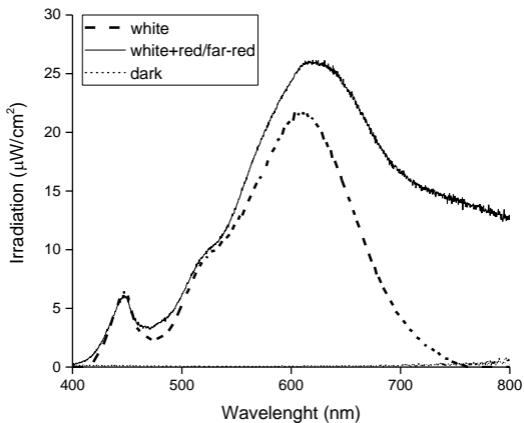

Supplement: Additional file 1: — Spectra of light treatments applied to bilberry fruits. (PDF 27 kb) [file 12870_2016_785_MOESM1_ESM.pdf]
